# Editorial

**Published:** 1844-05-18

**Authors:** 


### THE MEDICAL EXAMINER.

### PHILADELPHIA, MAY 18, 1844.

In the last number of the New York Journal of Medicine, in reply to some animadversions of our friend of the Bulletin of Medical Science, the Editor seeks to excuse himself by pleading our example; thus: "If there has been any *boasting*, it was commenced in the Philadelphia Examiner of the 27th January; and this demanded a reply in self-defence."—*N. Y. Journal*, May, 1844, page 426.

Our remarks, to which so much exception has been taken, were as follows: "The means of Clinical Instruction afforded by Philadelphia are yearly becoming more developed. With the exception of Paris and London, in no city perhaps, at this moment, is greater attention paid to Clinical Medicine, or better opportunities presented to students for acquiring a practical knowledge of the medical profession." Now if this be boasting, we cannot help it. As a journalist, we feel bound to state the case as it really is; and we believe most firmly, that, instead of exceeding, the above statement is even short of the truth. The opinion was not expressed at random, but after careful inquiry, and we have learned nothing since to alter, but much to confirm it. But let us see whether the *boasting* commenced in the Philadelphia Examiner. In the New York Journal of July 1843, page 142, we find the following statement: "The New York Hospital is *the largest of its class in this country*, having ample accomodation for at least three hundred and fifty patients. The diversity of diseases presented here is unsurpassed, *and, indeed*, unequalled, *by any similar institution either in this country or in Europe!*" Is there no boasting in that? Let it be remembered that this, with other like expressions contained in the same article, was published nearly seven months before those in the Examiner. By turning to the Examiner of the 4th instant, the reader will find an account of the New York Hospital, derived from the official Report of the Governors, by which he will be able to form his own opinion of the magnitude of that "*unsurpassed*" and "*unequalled*" Institution. Whether we are addicted to boasting or not, our readers must judge; but we think they will not often find in our pages such magniloquent terms as "*vast*," "*unlimited*," "*unsurpassed*," "*unequalled*," &c.

One more correction we must make, and then we shall close this subject for the present, hoping that we shall not have occasion to resume it. In the last number of the New York Journal, page iii. of the Editor's address to his Patrons, we find the following very erroneous and not very liberal statement; viz.: "The Philadelphia Hospital, or Blockley, as it is across the Schuylkill, and perhaps three miles distant, cannot be available for the purposes of clinical instruction. We refer here exclusively to the great body of students, who have not the advantage of extra courses of lectures."

What is meant by the last sentence we are at a loss to discover. Certainly all who visit the Hospital have the advantage of "extra courses of lectures," and that is just the reason why they go there. With regard to the statement that the Hospital is not available for clinical instruction, nothing can be further from the truth, and we are astonished that our brother of York should have fallen into the mistake. If he could be at the Colleges on the Wednesdays and Saturdays during the winter courses, and see the hundreds of students crowding into the omnibuses that convey them to the Hospital, or view them sitting for hours in its great amphitheatre, witnessing the operations that are performed, and listening to the able and instructive lectures that are there delivered, he would have more accurate knowledge on the subject. The Hospital is not "*three miles distant*;" but if it were, comfortable conveyance by carriages is provided without additional cost to the students, and they would be nothing loath to ride twice as far, we presume, on the same terms.

From the Report of the Auditors by whom the accounts of the Philadelphia Hospital were settled, for the year 1843, it appears that two thousand four hundred and twenty one dollars were received from students of medicine; and according to the Report of the Governors of the New York Hospital, page 6, the amount received at that institution for students, was eight hundred and nine dollars—just about one-third the amount at the Philadelphia Hospital.

Now for the *patients* in the two Institutions.

The number of medical and surgical cases in the Philadelphia Hospital, we are informed by the Secretary of the Managers, Mr. Robbins, is at no time *less* than three hundred, exclusive of the lunatics, and those in the children's asylum; the general average being from four to five hundred. The official report of the New York Hospital states, "the number of patients in the Institution on the 31st of December, 1842, was one hundred and ninety-eight." Where facts are at hand, there is little occasion for boasting.

